# Sulfated *Escherichia coli* K5 Polysaccharide Derivatives Inhibit Dengue Virus Infection of Human Microvascular Endothelial Cells by Interacting with the Viral Envelope Protein E Domain III

**DOI:** 10.1371/journal.pone.0074035

**Published:** 2013-08-28

**Authors:** Peter Vervaeke, Marijke Alen, Sam Noppen, Dominique Schols, Pasqua Oreste, Sandra Liekens

**Affiliations:** 1 Department of Microbiology and Immunology, Rega Institute for Medical Research, KU Leuven, Leuven, Belgium; 2 Glycores 2000 S.r.l., Milan, Italy; Queen's University, Canada

## Abstract

Dengue virus (DENV) is an emerging mosquito-borne pathogen that causes cytokine-mediated alterations in the barrier function of the microvascular endothelium, leading to dengue hemorrhagic fever (DHF) and dengue shock syndrome (DSS). We observed that DENV (serotype 2) productively infects primary (HMVEC-d) and immortalized (HMEC-1) human dermal microvascular endothelial cells, despite the absence of well-described DENV receptors, such as dendritic cell-specific intercellular adhesion molecule-3-grabbing non-integrin (DC-SIGN) or the mannose receptor on the cell surface. However, heparan sulfate proteoglycans (HSPGs) were highly expressed on these cells and pre-treatment of HMEC-1 cells with heparinase II or with glycosaminoglycans reduced DENV infectivity up to 90%, suggesting that DENV uses HSPGs as attachment receptor on microvascular endothelial cells. Sulfated *Escherichia coli* K5 derivatives, which are structurally similar to heparin/heparan sulfate but lack anticoagulant activity, were able to block DENV infection of HMEC-1 and HMVEC-d cells in the nanomolar range. The highly sulfated K5-OS(H) and K5-N,OS(H) inhibited virus attachment and subsequent entry into microvascular endothelial cells by interacting with the viral envelope (E) protein, as shown by surface plasmon resonance (SPR) analysis using the receptor-binding domain III of the E protein.

## Introduction

Dengue virus (DENV) is a mosquito*-*borne flavivirus that infects 50–100 million people each year, resulting in 500,000 hospitalizations and 25,000 deaths [1], [2]. Four closely related serotypes are known (DENV 1–4), which cause similar clinical manifestations, ranging from an acute self-limiting flu-like illness called dengue fever (DF) to the more severe and potentially life-threatening dengue hemorrhagic fever (DHF) and dengue shock syndrome (DSS). The latter are characterized by thrombocytopenia, increased capillary permeability and plasma leakage. Infection with one serotype leads to lifelong immunity against the same serotype, but only to partial and temporal immunity against the other serotypes [2]. Although a lot of research has been done on DENV, the pathogenesis of DHF/DSS is still not completely understood and no vaccine or antiviral treatment is available [2]–[5].

Dendritic cells, monocytes and macrophages are considered the primary targets of DENV infection *in vivo.* Several candidate receptors for DENV have been suggested on different cell types [6], including dendritic cell-specific intercellular adhesion molecule-3-grabbing non-integrin (DC-SIGN) on dendritic cells [7], the mannose receptor on macrophages [8] and the Fc-receptor on macrophages and monocytes after secondary infection with a heterologous serotype [4], [6]. Enhanced infection of immune cells, due to pre-existing non-neutralizing antibodies, and the resulting cytokine storm have been suggested to be involved in DHF/DSS development [3], [4].

However, direct infection of endothelial cells may be an additional factor contributing to DENV-increased vascular permeability. The presence of DENV-infected endothelial cells was demonstrated in murine models, and DENV antigens were found in endothelial cells in patient autopsy samples [9]–[14]. *In vitro*, DENV has been shown to replicate in primary macrovascular endothelial cells and in endothelial cell lines of macrovascular and microvascular origin [14]–[20]. Microarray analysis of DENV-infected endothelial cells revealed several differentially expressed genes involved in stress, cell adhesion, wounding, inflammatory and antiviral pathways [21]–[23]. In particular, infection of human endothelial cells with DENV resulted in transcriptional up-regulation and secretion of various cytokines, including interleukin (IL)-6, IL-8 and tumor necrosis factor (TNF)-α [24]–[28], which are also highly upregulated in the sera of patients with DHF and may contribute to increased endothelial cell permeability [27], [29]. Increased vascular permeability of HMEC-1 cells infected with DENV was associated with actin reorganization and alterations in the expression and distribution of adherens and tight junctional proteins involved in endothelial cell integrity [20], [26]. Also DENV-induced changes in surface and soluble vascular endothelial growth factor (VEGFR)-2 expression were implicated in plasma leakage in DHF [30]. Moreover, DENV infection was shown to alter the production of various coagulation factors in endothelial cells, including increased expression of thrombomodulin [31]–[33], downregulation of the cytoprotective protein C pathway [34], reduced secretion of tissue plasminogen activator (tPA) [25], [31] and upregulated expression of E-selectin, resulting in increased adherence of platelets to the infected endothelial cells [35]. Thus, various mechanisms may act in concert in DENV-infected endothelial cells to contribute to the vascular leakage and thrombocytopenia observed in DSS.

However, the target receptor for DENV in endothelial cells remains to be identified and inhibitors of endothelial cell infection by DENV have not yet been reported. Zhang *et al.* [36] proposed β3 integrin as a potential DENV receptor and recently, Dalrymple and Mackow [17] reported that heparan sulfate proteoglycans (HSPGs) are important for DENV infection in human umbilical vein endothelial cells (HUVEC). Since DENV-induced plasma leakage occurs at the level of the microvasculature [37], [38] we studied DENV infection in primary human dermal microvascular endothelial cells (HMVEC-d) and the microvascular cell line HMEC-1 [39]. We found that DENV infection of microvascular endothelial cells is mediated by HSPGs. Therefore, we investigated whether heparin analogues display anti-DENV activity in these cells.

Many polyanionic compounds have been developed over the years to block virus entry. However, the clinical use of these heparin analogues has been limited because of anticoagulant side-effects. Chemically sulfated derivatives of the K5 capsular polysaccharide of *Escherichia coli* emerged as a promising new class of antivirals, with activity against human immunodeficiency virus (HIV) [40], herpes simplex viruses (HSV) [41], human papillomaviruses (HPVs) [42] and human cytomegalovirus (HCMV) [43]. These compounds are synthesized from a polysaccharide that has the same structure as the biosynthetic precursor of heparin/heparan sulfate, N-acetyl heparosan [44], but are devoid of toxicity and anticoagulant activity [45]. We demonstrate that the highly sulfated K5-OS(H) and K5-N,OS(H) inhibit DENV attachment and entry in microvascular endothelial cells by interacting with domain III of the viral envelope protein, indicating that these agents may represent a promising new class of anti-DENV agents.

## Materials and Methods

### Cell lines and virus

The human microvascular endothelial cell line HMEC-1 [39] was obtained from the Centers for Disease Control and Prevention (CDC, Atlanta, GA, USA) and was grown in Dulbecco’s Modified Eagle Medium (DMEM, Invitrogen, Merelbeke, Belgium) supplemented with 10% fetal bovine serum (FBS, Integro, Dieren, The Netherlands), 0.01 M HEPES (Invitrogen) and 1 mM sodium pyruvate (Invitrogen) at 37°C under 5% CO_2_. Only cells with passage number 20 to 25 were used. Primary human dermal microvascular endothelial cells (HMVEC-d) were purchased from Lonza (Verviers, Belgium) and grown in microvascular endothelial cell growth medium (EGM MV, Lonza) at 37°C under 5% CO_2_. C6/36 mosquito cells from *Aedes albopictus* (ATCC) were maintained at 28°C and grown in Minimum Essential Medium (MEM, Invitrogen) supplemented with 10% FBS, 0.01 M HEPES, 2 mM L-glutamine (Invitrogen) and non-essential amino acids (Invitrogen). Baby hamster kidney (BHK) cells were kindly provided by Dr. M. Diamond (Washington University, St Louis, USA). These cells were used for virus titration and were grown in DMEM supplemented with 10% FBS, 0.01 M HEPES and 1 mM sodium pyruvate at 37°C under 5% CO_2_.

DENV serotype 2 laboratory-adapted New Guinea C (NGC) strain (DENV-2), a kind gift from Dr. V. Deubel (Institut Pasteur, Paris, France), was propagated in C6/36 cells. Supernatant containing the virus was collected 5 days post-infection and stored at –80°C. Virus was titrated by the plaque assay using BHK cells. Ten-fold dilutions of the virus were inoculated on monolayers of BHK cells cultured in a 6-well plate (International Medical Products, Watermaal-Bosvoorde, Belgium) for 4 h at 37°C. The cells were washed two times with growth medium and incubated with an overlay containing 1% Avicel [46] and DMEM with 2% FBS in a 1:1 ratio. After 4 days the cells were washed with PBS, fixed with 70% ethanol and stained with 0.1% crystal violet. Plaques were counted to determine the viral titer in plaque forming units per ml (PFU/ml).

### Chemicals and antibodies

Heparin, heparan sulfate, chondroitin sulfate A, dermatan sulfate, BSA and heparinase II were purchased from Sigma-Aldrich. *Escherichia coli* K5 polysaccharide derivatives (K5, K5-NS, K5-OS(L), K5-OS(H), K5-N,OS(L) and K5-N,OS(H)) were synthesized as described before [47]. The mannose-specific plant lectin from *Hippeastrum hybrid* (HHA) (50 kD) was derived and purified from these plants as described previously [48]. Cilengitide, a cyclic RGD peptide, was kindly provided by Dr. H. Kessler (Technische Universität München, München, Germany). The monoclonal antibody against DENV-2 envelope (E) glycoprotein (clone 3H5) and functional blocking α_v_β_3_-integrin antibody (clone LM609) were purchased from Chemicon International/Millipore (Billerica, MA, USA) and secondary phycoerythrin-conjugated goat anti-mouse antibody (clone Poly4053) was from Imtec Diagnostics (Antwerpen, Belgium). Heparan sulfate antibody (10E4 epitope) was obtained from Immunosource (Zoersel, Belgium). Anti-vascular endothelial growth factor receptor-2 (VEGFR-2) and anti-platelet endothelial cell adhesion molecule-1 (PECAM-1/CD31) were purchased from Abcam (Cambridge, UK), mouse anti-β3 integrin antibody was from Santa Cruz Biotechnology (Heidelberg, Germany). DC-SIGN, liver/lymph node-specific intercellular adhesion molecule-3-grabbing integrin (L-SIGN) and mouse IgG_2B_ isotype control antibodies were obtained from R&D Systems (Minneapolis, MN, USA). The mannose receptor antibody (clone 19.2) and mouse IgG_2a_ isotype control antibody (clone MOPC-173) were from BD Biosciences (Erembodegem, Belgium).

### Flow cytometric analysis of endothelial cell surface markers

HMEC-1 and HMVEC-d cells were detached by exposure to non-enzymatic cell dissociation solution (Sigma-Aldrich, Bornem, Belgium), washed twice with PBS containing 2% FBS, and incubated at room temperature for 45 min with primary antibody or FITC-/PE-conjugated antibodies directed against the surface markers of interest. Next, cells were washed with 2% FBS/PBS and incubated at room temperature for 30 min with PE- or FITC-labeled secondary antibodies. Cells incubated with labeled secondary antibody only or corresponding FITC-/PE-conjugated isotypic control antibodies were used as negative control. Finally, cells were washed twice with 2% FBS/PBS, fixed with 2% paraformaldehyde (PFA, Sigma-Aldrich) in PBS and analyzed by flow cytometry.

### DENV infection of endothelial cells and monocyte-derived dendritic cells (MDDC)

HMEC-1 or HMVEC-d cells (1×10^5^ cells/well) were seeded in a gelatin-coated 48-well plate (International Medical Products). After an overnight incubation, confluent monolayers were infected with DENV-2 at a multiplicity of infection (MOI) of 1 (unless otherwise stated) for 4 h at 37°C (in the presence or absence of compound). Unbound virus was removed by washing twice with growth medium after which the cells were incubated at 37°C in fresh culture medium without compounds. Cells were analyzed for DENV infection by flow cytometry at different time points and the supernatant was stored at –80°C for viral RNA extraction and real-time RT-PCR analysis.

MDDC were isolated and infected with DENV-2 as described previously by Alen *et al.* [49].

### Flow cytometric analysis of DENV-infected cells

HMEC-1, HMVEC-d or MDDC cells were washed with 2% FBS/PBS, fixed and permeabilized using the Cytofix/Cytoperm Kit (BD Biosciences, San Diego, CA, USA) according to the manufacturer’s instructions. The permeabilized cells were stained with anti-DENV-2 E protein antibody for 30 min on ice, washed and incubated for 30 min on ice with the secondary PE-conjugated goat anti-mouse antibody. The stained cells were analyzed by flow cytometry (FACSCalibur, BD Biosciences, San Jose, CA, USA). Data were analyzed with CellQuest software (BD Biosciences).

### RNA extraction and real-time RT-PCR

Viral RNA was extracted from 150 µl of supernatant with the Nucleospin® RNA Virus Kit (Macherey-Nagel, Düren, Germany) according to the manufacturer’s instructions. RT-PCR was performed as described previously [49] using the ABI 7500 Fast Real-Time PCR System (Applied Biosystems, Branchburg, NJ, USA). Data were analyzed with ABI PRISM 7500 software (version 2.0.5, Applied Biosystems).

### Virus yield reduction assay

BHK monolayers grown in 6-well plates were incubated with 10-fold serial dilutions of supernatant from HMEC-1 cells infected with DENV-2 in the presence or absence of compound (300 nM). After 4 h, the medium was removed and the cells were incubated with Avicel overlay for 4 days after which the plaques were counted.

### Time-of-drug addition assay

HMEC-1 cells were infected as described above. K5-OS(H) and K5-N,OS(H) were added 30 min prior to, at the moment of, or at different times post-infection. Compounds were also incubated for 30 min with the DENV-2 inoculum before infection. Four hours after infection, unbound virus and compounds were removed and cells were incubated with fresh growth medium for 24 h. DENV infection of the cells was analyzed by flow cytometry.

### Attachment assay

DENV-2 and different concentrations of K5 derivatives were added to HMEC-1 monolayers at 4°C for 2 h. Next, cells were washed twice (at 4°C) to remove unbound virus and compound, incubated for 24 h in growth medium without compound at 37°C and analyzed by flow cytometry.

For immunofluorescence staining, HMEC-1 cells (5×10^4^ cells/well) were seeded in 8-well Lab-Tek chamber slides (Nalge Nunc International, Roskilde, Denmark). After 24 h, cells were infected with DENV-2 at a MOI of 10 in the presence or absence of compounds for 1 h at 4°C. Unbound virus was removed by washing 3 times with ice-cold PBS and cells were fixed with 2% PFA for 15 min at room temperature. Fixed cells were blocked with 0.5% BSA in PBS for 1 h at room temperature, washed twice with PBS and incubated with anti-DENV-2 E protein antibody for 2 h. Goat anti-mouse IgG Alexa Fluor 488 (Invitrogen) was used as secondary antibody, nuclei were stained with DAPI (Invitrogen). Fluorescence analysis was performed on an Axiovert 200 M microscope (Carl Zeiss, Götingen, Germany) using an EC Plan-Neofluar 40X/1.3 oil objective.

### Entry assay

DENV-2 was added to HMEC-1 monolayers at 4°C for 2 h to allow virus attachment. Next, cells were washed twice to remove unbound virus and incubated with growth medium containing different concentrations of K5 derivatives at 37°C for 2 h to allow virus entry. Cells were washed twice and treated with citrate buffer (citric acid 40 mM, potassium chloride 10 mM, sodium chloride 135 mM, pH 3) for 1 min to inactivate adsorbed but not internalized virus. Next, cells were incubated for 24 h in growth medium without compound at 37°C and analyzed by flow cytometry.

### Cytotoxicity assay

Potential cytotoxic effects of the compounds were evaluated by the MTS method [3-(4,5-dimethylthiazol-2-yl)-5-(3-carboxymethoxyphenyl)-2-(4-sulfophenyl)-2H-tetrazolium; Promega, Leiden, The Netherlands)]. HMEC-1 cells were seeded at 5×10^3^ cells/well in a 96-well plate. After an overnight incubation, growth medium was removed and cells were incubated with serial dilutions of the compounds for 48 h. Next, medium was replaced by 100 µl of 0.02% MTS in PBS and incubated for 2 h at 37°C. The optical density (OD) was determined at 490 nm and the 50% cytotoxic concentration (CC_50_; i.e. drug concentration needed to reduce the total cell number by 50%) was calculated.

### Heparinase II treatment

Confluent monolayers of HMEC-1 cells were incubated with heparinase II (0.5 to 10 Sigma Units/ml diluted in PBS) for 2 h at 37°C to selectively remove glycosaminoglycan (GAG) chains. The cells were subsequently infected as described above. To confirm cleavage by heparinase II, cells were collected and stained with a mouse monoclonal anti-heparan sulfate antibody, washed with 2% FBS/PBS, incubated with a secondary PE-labeled goat anti-mouse antibody and analyzed by flow cytometry.

### Surface plasmon resonance (SPR) analysis

Recombinant envelope protein (domain III) of DENV-2 (amino acid sequence: SYSMCTGKFKIVKEIAETQHGTIVIRVQYE GDGSPCKIPFEIMDLEKRHVLGRLITVNPIVTEKDSPVNIEAEPPFGDSYIIIGVEPGQLKLDWFKKGSSIGQMFETTMRGAKRMA) (GenWay Biotech Inc., San Diego, CA, USA) was covalently immobilized on a CM5 sensor chip in 10 mM sodium acetate, pH 5.5, using standard amine coupling chemistry. The chip density was 1800 resonance units (RU). A reference flow cell was used as a control for non-specific binding and refractive index changes. All interaction studies were performed at 25°C on a Biacore T200 instrument (GE Healthcare, Uppsala, Sweden). The compounds K5, K5-OS(H) and K5-N,OS(H) were serially diluted in HBS-P (10 mM HEPES, 150 mM NaCl and 0.05% surfactant P20; pH 7.4) and 10 mM CaCl_2_ covering a concentration range between 12 nM and 1.5 µM, by using five-fold dilution steps. Anti-DENV-2 E antibody and mouse IgG_2B_ isotype control antibody were included as positive and negative control, respectively. Samples (in duplicate) were injected for 2 min at a flow rate of 45 µl/min and the dissociation was followed for 3 min. Several buffer blanks were used for double referencing. The CM5 sensor chip surface was regenerated with a single injection of 50 mM NaOH. For kinetic analysis the same experimental setup was applied but with a chip density of 680 RU. The experimental data were fit using the 1:1 binding model (Biacore T200 Evaluation software 1.0) to determine the binding kinetics.

To evaluate competition between recombinant E protein domain III and K5 derivatives for binding to heparin, 30 RU of biotinylated heparin (Sigma) were captured on a streptavidin (SA) sensor chip. The analytes, 400 nM of E protein domain III alone or premixed with K5 derivatives or heparin (at different concentrations), were diluted in HBS-EP (10 mM HEPES, 150 mM NaCl, 3 mM EDTA and 0.05% surfactant P20; pH 7.4). Samples were injected for 2 min at a flow rate of 30 µl/min, followed by a dissociation phase of 2 min. The SA sensor chip surface was regenerated with a single injection of 50 mM NaOH and washed with buffer for 6 min.

## Results and Discussion

### 

#### Microvascular endothelial cells are permissive for DENV-2 infection and replication

The microvascular endothelium forms the primary fluid barrier of the capillaries, but the extent to which the endothelial cells contribute to increased capillary permeability in DHF/DSS is not well understood [37], [38]. However, it is likely that infected endothelial cells contribute to increased viral spread and that viral antigens expressed by these cells target the endothelium for immune attack. As such, it is important to gain further insight into the mechanism by which DENV infects endothelial cells and to search for ways to interfere with this process.

**Figure 1:**
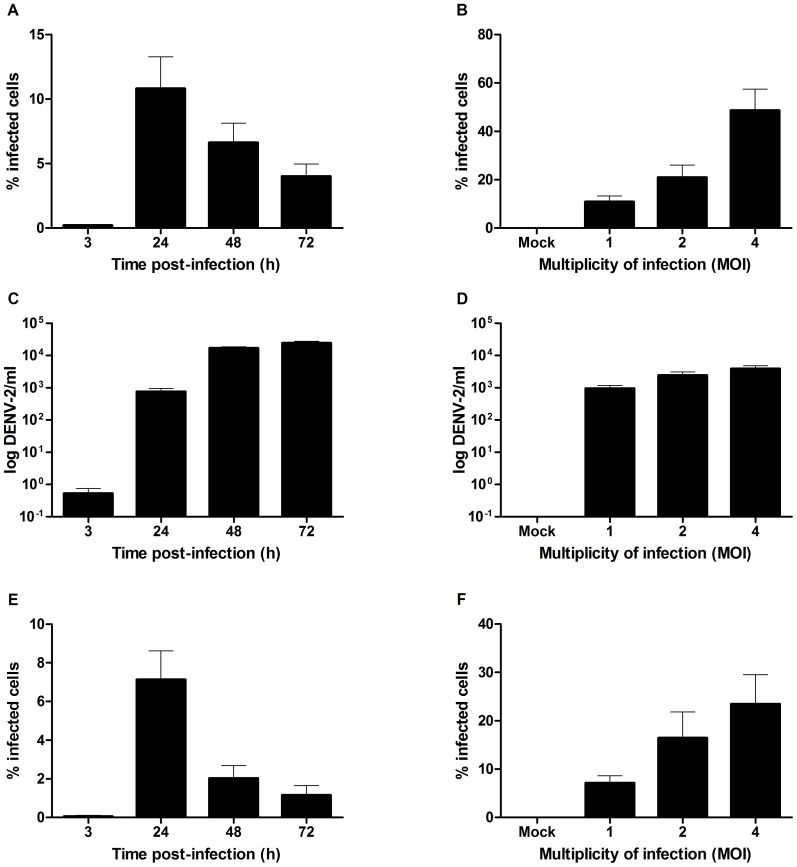
Time- and dose-dependent DENV infection of primary microvascular endothelial HMVEC-d cells and the endothelial cell line HMEC-1. HMEC-1 (A, C) and HMVEC-d cells (E) were infected with DENV-2 at a MOI 1. Viral infectivity was quantified at different times after infection by flow cytometry using an anti-DENV-2 specific antibody (A, E). The amount of viral RNA was determined in the supernatant of infected HMEC-1 cells by means of real-time RT-PCR at different times after infection (C). Alternatively, HMEC-1 (B, D) and HMVEC-d cells (F) were treated with medium only (mock) or infected with DENV at a MOI of 1, 2 or 4. Viral infectivity was quantified 24 h post infection by flow cytometry (B, F). The amount of viral RNA was determined in the supernatant of infected HMEC-1 cells at 24 h post infection by means of real-time RT-PCR (D). The means and standard deviations of three independent experiments are shown.

We investigated DENV infection and replication in microvascular endothelial cells by means of flow cytometry and RT-PCR at different time points post-infection. When HMEC-1 cells were infected with DENV-2 at a MOI of 1, 11% of the cells were infected at 24 h post-infection (Fig. 1A). This number could be increased up to 49% when a MOI of 4 was used (Fig. 1B). Histograms representing flow cytometric analyses are shown in Fig. S1. The infected cells were susceptible to DENV replication as shown by the increase in viral load 24-48 h post-infection (Fig. 1C, D).

These levels of infection are in agreement with a previous study by Zamudio-Meza *et al.* [16], who reported a productive infection in 25% of HMEC-1 cells infected with DENV-2 at an MOI of 3 (compared with 21% infection obtained at an MOI of 2 in our study). Dalrymple and Mackow [17] obtained an infection rate of more than 80% in primary endothelial cells (human umbilical vein endothelial cells; HUVECs). However, these studies were performed with DENV serotype 4, whereas we used DENV serotype 2. Additionally, HUVECs are macrovascular endothelial cells and it is well-established that the functional differences that exist between large and small vessels are reflected by morphological and phenotypic differences between macrovascular and microvascular endothelium [50], including the expression of specific surface markers, which may ultimately determine DENV attachment and entry.

To our knowledge, DENV infection in primary microvascular endothelial cells has never been studied. Therefore, we decided to investigate whether human primary microvascular endothelial cells of dermal origin (HMVEC-d) are susceptible to DENV-2 infection. Under the same experimental conditions as used for HMEC-1 cells, 7% to 23% of HMVEC-d cells were infected with DENV at a MOI of 1 or 4, respectively (Fig. 1 E, F). This suggests that multiple parameters, including origin of the cells, passage number, macrovascular versus microvascular endothelium, may affect DENV infectivity in endothelial cells. Indeed, significant differences in DENV infection rate and cell surface expression of adhesion molecules were demonstrated using microvascular endothelial cell lines of liver and dermal origin [19]. Also the DENV NS1 protein, which is secreted from infected cells, was shown to preferentially bind to cultured human microvascular compared to aortic or umbilical vein endothelial cells [51].

However, the decrease in the number of infected HMEC-1 and HMVEC-d cells that was observed after 24 h and the stabilization of virus titer after 48–72 h (Fig. 1) correlate with the findings obtained in HUVECs [23] and were suggested to be the result of an IFN-β response induced in DENV-infected endothelial cells, which protects the neighboring cells from DENV infection. Therefore, in the following experiments we preferred to investigate viral infectivity and replication after 24 h and 48 h, respectively.

### Expression of putative DENV-2 receptors on microvascular endothelial cells

**Figure 2:**
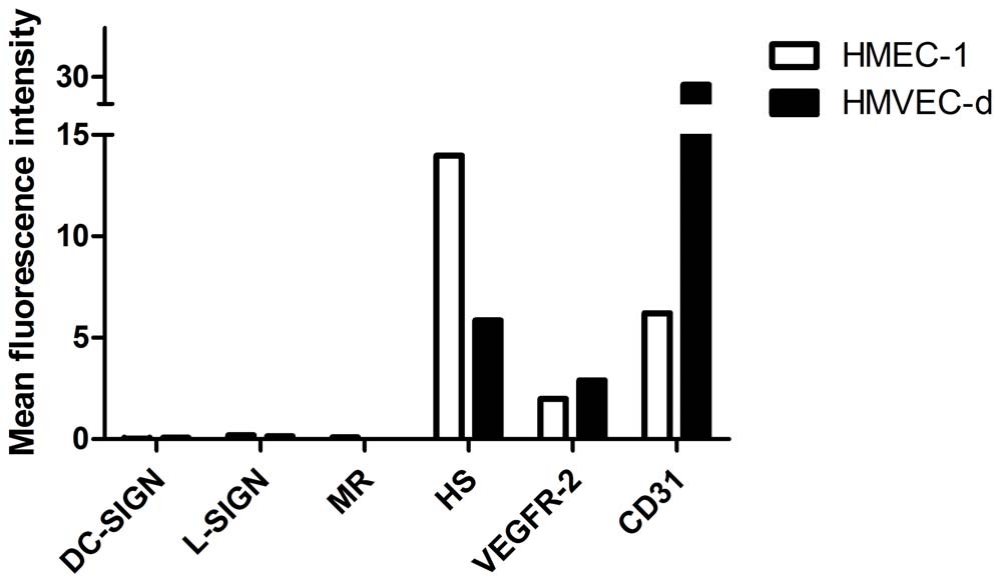
Expression of surface molecules on HMEC-1 and HMVEC-d cells as determined by flow cytometry. HMEC-1 and HMVEC-d cells were stained with antibodies against different DENV receptors or endothelial cell markers. The mean fluorescence intensities for DC-SIGN, L-SIGN, mannose receptor (MR), heparan sulfate (HS), vascular endothelial growth factor receptor (VEGFR)-2 and CD31 (PECAM-1) are shown (after background reduction in the presence of labeled secondary antibody only or corresponding FITC-/PE-conjugated isotypic control antibody).

DENV has been shown to infect various cell types by binding to different receptors, i.e. DC-SIGN (CD209) on dendritic cells, the mannose receptor (CD206) on macrophages and the Fc-receptor on monocytes and macrophages [6]–[8], but the receptor for DENV on endothelial cells has not yet been revealed. DC-SIGN and the mannose receptor were not detected on the surface of HMEC-1 and HMVEC-d cells (Fig. 2). Also, these cells did not express L-SIGN, which is found on endothelial cells of liver and lymph nodes [52]. Other typical endothelial cell markers, such as PECAM-1 (CD31) and vascular endothelial growth factor receptor (VEGFR)-2 could be detected on the endothelial cells. However, it should be noted that primary HMVEC-d cells expressed higher levels of CD31, compared with the HMEC-1 cell line, whereas both cell types expressed equally (low) levels of VEGFR-2 (Fig. 2).

Zhang *et al.* [36] proposed β3 integrin as a potential receptor for DENV on endothelial cells. Although we confirmed that this integrin is present on HMEC-1 cells (Fig. S2), pre-treatment of the cells with a functional blocking antibody directed at β_3_ integrin or with Cilengitide, a RGD-containing peptide [53], did not abrogate DENV infection (Fig. S2), suggesting that β_3_ integrin does not mediate DENV infection in microvascular endothelial cells.

Recently, Dalrymple and Mackow [17] showed that HSPGs mediate DENV serotype 4 infection in HUVECs. We found that HSPGs are highly expressed on the surface of HMEC-1 and HMVEC-d cells (Fig. 2). Also, HSPGs have been reported to mediate DENV infection in various other cell types (e.g. Vero monkey kidney cells, BHK cells, and several human liver cell lines) [54]–[59]. Hence, the potential role of HSPGs in DENV-2 infection of microvascular endothelial cells was further investigated.

### DENV infection of HMEC-1 cells is mediated by heparin-containing glycosaminoglycans (GAGs)

**Figure 3:**
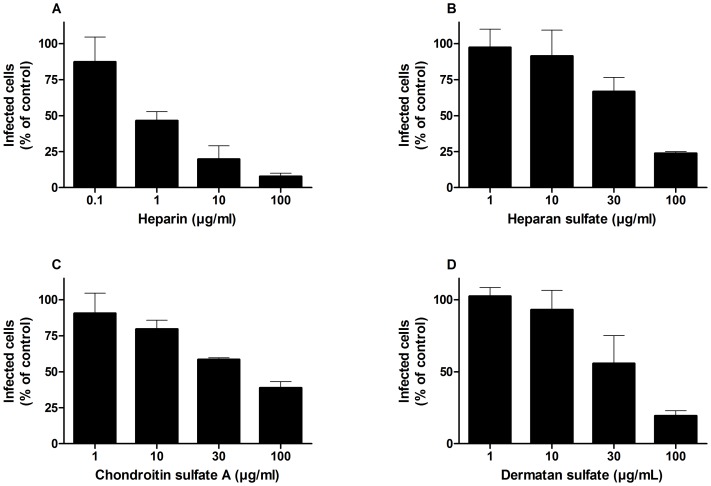
Anti-DENV-2 activity of sulfated GAGs. HMEC-1 cells were infected with DENV-2 at a MOI 1. Viral infectivity was quantified 24 h after infection by flow cytometry using an anti-DENV-2 specific antibody. Heparin (A), heparan sulfate (B), chondroitin sulfate A (C) and dermatan sulfate (D) dose-dependently inhibited DENV-2 infection in HMEC-1 cells. Data represent the % of infected cells relative to the positive control (DENV-2 infected cells). The means and standard deviations of three independent experiments are shown.

HSPG receptors are composed of a core protein and different GAG side-chains [60]. Therefore, the anti-DENV activity of naturally occurring GAGs (heparin, heparan sulfate, chondroitin sulfate A and dermatan sulfate) was investigated. A dose-dependent inhibitory activity was observed for all four GAGs by flow cytometry for viral antigen expression (Fig. 3). EC_50_ values were determined for each compound and are shown in Table 1. Heparin showed the most pronounced antiviral activity with an EC_50_ value (i.e. compound concentration that inhibits DENV infection by 50%) of 77 nM and could inhibit DENV-2 infection of HMEC-1 cells up to 93% at the highest concentration tested (7.7 µM). Heparan sulfate, chondroitin sulfate A and dermatan sulfate, in contrast, only showed antiviral activity (35–80%) at micromolar concentrations (3–6 µM).

**Table 1:** Antiviral activity of heparin and heparan sulfate analogs against DENV-2 in HMEC-1 cells.

Compound	EC_50_ (µg/mL)	EC_50_ (µM)
Heparin	1.0±0.3	0.077±0.023
Heparan sulfate	60±10	6.0±1.0
Chondroitin sulfate A	67±5	4.2±0.3
Dermatan sulfate	46±17	3.3±0.9

EC_50_: effective concentration or drug concentration required to inhibit DENV-2 infection in HMEC-1 cells by 50% as measured by flow cytometry. The means ± standard deviations for three independent experiments are shown.

**Figure 4:**
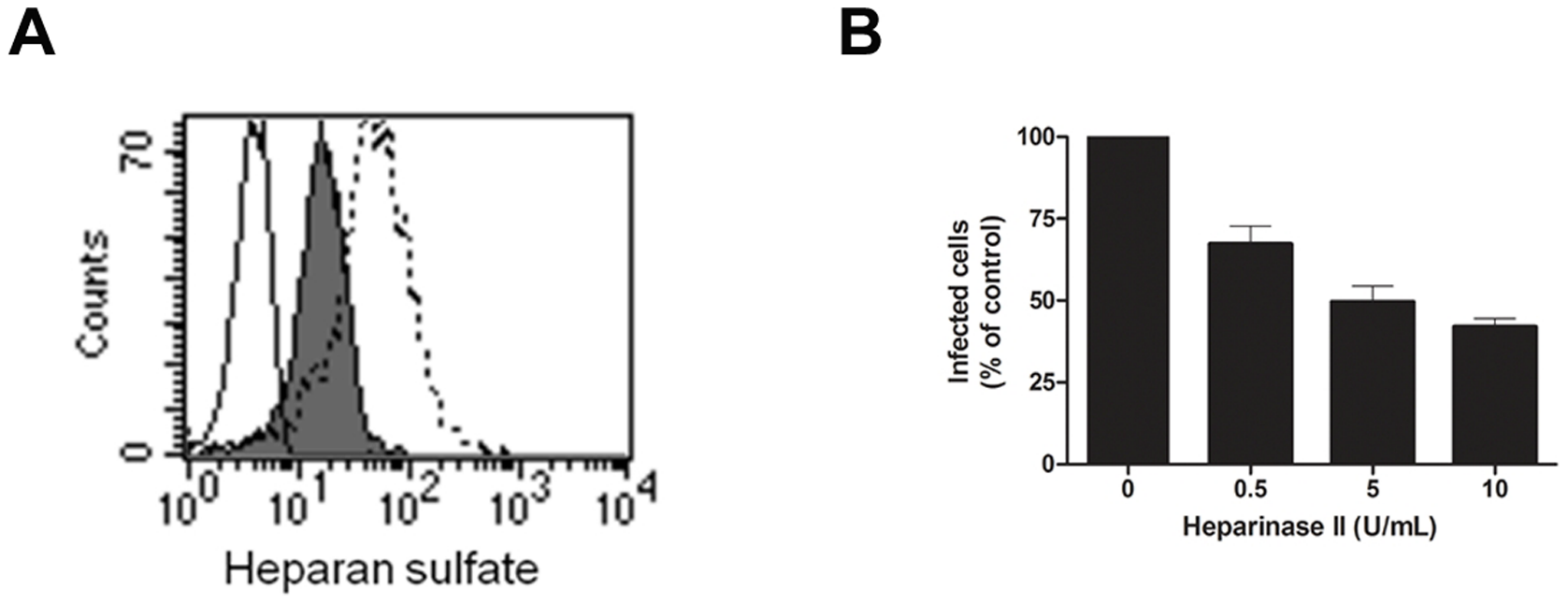
Heparinase II treatment of HMEC-1 cells reduces DENV-2 infectivity . HMEC-1 cells were treated with 10 U/mL of heparinase II (A). Cell surface expression of heparan sulfate was analyzed by flow cytometry and plotted against the number of events (counts). The dashed line represents untreated HMEC-1 cells, the full histogram indicates heparinase II-treated HMEC-1 cells. Background in the absence of primary antibody is shown by the full line. HMEC-1 cells were treated with different concentrations of heparinase II (0 to 10 U/mL) and subsequently infected with DENV-2 at a MOI 1 (B). Viral infectivity was quantified by flow cytometry using an anti-DENV-2 specific antibody. The means and standard deviations of three independent experiments are shown.

To further analyze the role of HSPGs in DENV-2 infection of microvascular endothelial cells, HMEC-1 cells were treated with different concentrations of heparinase II, which cleaves sulfated polysaccharide chains containing 1–4 linkages between uronic acid and hexosamine residues. Thus, the enzyme is able to cleave heparan sulfate and to a lesser extent heparin (relative activity ∼ 2:1) [61]. Incubation with 10 U/ml heparinase II led to a decline of 70% in heparan sulfate surface expression (Fig. 4A) and a 58% reduction in the number of infected cells (Fig. 4B). As treatment with heparinase II had no effect on cell viability, the reduced number of infected cells was not caused by toxic effects on HMEC-1 cells. Pretreatment of endothelial cells with chondroitinase ABC (which cleaves dermatan sulfate and chondroitin sulfate) had no effect on DENV-2 infection of HMEC-1 cells (data not shown). These data suggest that DENV-2 infection of HMEC-1 cells specifically requires the interaction of DENV with heparin.

### Inhibition of DENV infection by *Escherichia coli* K5 polysaccharide derivatives

Many polyanions and heparin analogs have been investigated for their use as antiviral drugs over the past decades but their clinical use has been limited because of anticoagulant activity and poor oral bioavailability [62]. Sulfated derivatives of the K5 polysaccharide of *Escherichia coli* contain a backbone with the same structure as the biosynthetic precursor of heparin and HS, but are devoid of anticoagulant activity [45]. Therefore, several K5 derivatives (i.e. the native form K5, N-sulfated K5 (K5-NS), O-sulfated K5-OS(L) and N,O-sulfated K5-N,OS(L) with low degree of sulfation and K5 with high degree of sulfation K5-OS(H) and K5-N,OS(H), see Table 2), were evaluated for their antiviral activity against DENV-2 in microvascular endothelial cells.

**Table 2:** Chemical features of sulfated K5 derivatives.

Compound	MW(kDa)	SO_3_^−^/COO^−^ ratio	Chemical composition (%)				
			Glc-NSO_3_^−^	Glc-6SO_3_^−^	GlcA-OSO_3_^−^	GlcA2,3SO_3_^−a^	Nonsulfated GlcA
K5	30	0	0	0	0	0	100
K5-NS	15	1.00	100	0	0	0	100
K5-OS(L)	14	1.41	0	90	<10	0	>90
K5-OS(H)	11	3.77	0	100	0	100	0
K5-N,OS(L)	13	1.70	100	90	<10	0	>90
K5-N,OS(H)	15	3.84	100	100	30	70	0

a GlcA2SO_3_^−^ or GlcA3SO_3_^−^.

**Figure 5:**
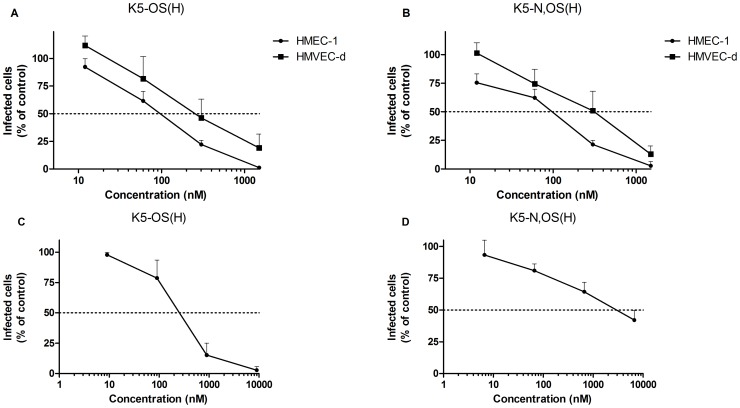
Dose-dependent antiviral activity of K5-OS(H) and K5-N,OS(H) in DENV-infected endothelial cells and MDDC. HMEC-1 or HMVEC-d cells (A, B) and MDDC (C, D) were infected with DENV-2 at a MOI 1 in the presence of different concentrations of K5-OS(H) (A, C) or K5-N,OS(H) (B, D). Viral infectivity was quantified 24 h (endothelial cells) or 48 h (MDCC) post infection by flow cytometry using an anti-DENV-2 specific antibody. Data represent the % of infected cells relative to the virus control. The means and standard deviations of three independent experiments are shown.

**Figure 6:**
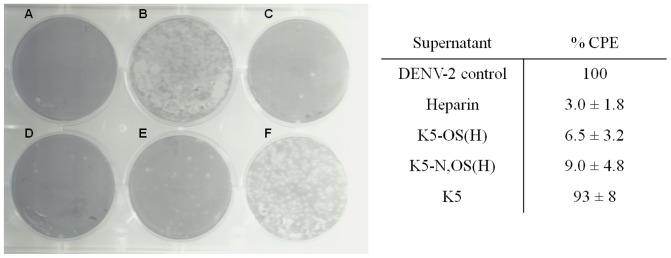
Virus yield reduction assay on BHK cells. HMEC-1 cells were infected with DENV-2 (MOI 1) in the presence or absence of the compounds (300 nM). After 24 h, supernatant was collected and transmitted to a monolayer of BHK cells at serial 10-fold dilutions. Plaques were counted after 4 days. Left: A representative virus yield reduction assay of dilution 10^−1^ is shown: Cell control (A), virus control (B), heparin (C), K5-OS(H) (D), K5-N,OS(H) (E) and K5 (F). Right: The % cytopathic effect (CPE) relative to the virus control ± standard deviation is shown.

K5 and K5-NS did not inhibit DENV-2 infection even at the highest concentration tested (3 µM), while K5-OS(L) and K5-N,OS(L) only inhibited DENV-2 infection by 42 and 19%, respectively, when administered at 3 µM, but lost activity at 5-fold lower concentrations. Instead the highly sulfated K5-OS(H) and K5-N,OS(H) proved to be very active compounds, which dose-dependently inhibited DENV-2 infection in the nanomolar range, with an EC_50_ value of 113±80 and 111±50 nM, respectively (Fig. 5, Table 3). The antiviral activity of the K5 derivatives was not due to cytotoxic effects (CC_50_ > 10 µM), resulting in a favorable selectivity index (SI) of 176 and 113 for K5-OS(H) and K5-N,OS(H), respectively (Table 3). Both compounds suppressed DENV-2 replication (measured by RT-PCR) with an EC_50_ of 168±88 and 201±57 nM, respectively (data not shown). To study the effect of sulfated K5 derivatives on infectious virus production, a virus yield reduction assay was performed in BHK cells using supernatant from HMEC-1 cells, infected with DENV in the presence (300 nM) or absence of the compounds. As expected, heparin (97%), K5-OS(H) (94%) and K5-N,OS(H) (91%), but not K5 (7%) significantly reduced the formation of plaques induced by infectious virus released from HMEC-1 cells (Fig. 6).

**Table 3:** Antiviral activity and cytotoxicity of K5 derivatives in HMEC-1 cells.

Compound	MW (kDa)	CC_50_ (µM)	EC_50_ (µM)	SI
**K5**	30	10±2	> 3	
**K5-NS**	15	> 50	> 3	
**K5-OS(L)**	14	> 50	> 3	
**K5-OS(H)**	11	20±2	0.11±0.08	176
**K5-N,OS(L)**	13	17±2	> 3	
**K5-N,OS(H)**	15	13±1	0.11±0.05	113

CC_50_: cytotoxic concentration or drug concentration required to reduce cell viability by 50% as measured by MTS assay.

EC_50_: effective concentration or drug concentration required to inhibit DENV-2 infection in HMEC-1 cells by 50% as measured by flow cytometry. The means ± standard deviations for three independent experiments are shown.

SI: selectivity index, ratio of CC_50_/EC_50._

Next, we investigated whether the compounds also display antiviral activity in primary HMVEC-d cells. Again, K5-OS(H) and K5-N,OS(H) dose-dependently inhibited DENV-2 infection (EC_50_ value of 266 and 330 nM, respectively) (Fig. 5), whereas the other K5 derivatives were ineffective (data not shown).

Together, these data indicate that the highly sulfated K5 derivatives inhibit the interaction of DENV with HSPGs on endothelial cells. As such, the 3-fold lower activity of the compounds in HMVEC-d cells, compared with HMEC-1 cells, may be explained by the lower expression of HSPGs in the primary ECs, as shown by flow cytometric analysis (Fig. 2). Our findings also suggest that the antiviral activity of the K5 derivatives depends on their degree of sulfation, but not on the position of the sulfate groups (O *versus* N). The requirement for highly sulfated compounds may in part explain why heparin was almost 100-fold more effective than heparan sulfate in inhibiting DENV infection of endothelial cells. Alternatively, the composition of the disaccharide unit may explain the marked difference in antiviral activity of these GAGs; whereas heparan sulfate is composed of glucuronic acid (linked to N-acetylglucosamine), heparin contains mainly iduronic acid. The latter hypothesis suggests that the anti-DENV activity of K5 derivatives may be improved by replacing glucuronic acid with iduronic acid [63].

Since dendritic cells are considered the primary target cells of DENV *in vivo*, we next investigated whether the compounds display antiviral activity in monocyte-derived dendritic cells (MDCC). As a positive control, we used the carbohydrate-binding agent HHA, which has been shown to interrupt the interaction of DENV with DC-SIGN, the cognitive receptor for DENV on MDDC [49]. HHA displayed a potent inhibition of DENV infection in MDCC (EC_50_ value of 5.6±2.8 nM). Interestingly, also K5-OS(H) and K5-N,OS(H) impaired DENV-2 infection of MDCC (Fig. 5), although to a lesser extent than HHA. However, whereas K5-OS(H) almost completely retained antiviral activity in MDDC (EC_50_ value of 291±85 nM versus 113±80 nM in endothelial cells), K5-N,OS(H) was less effective (EC_50_ value of 2.6±1.1 µM).

We found high expression of DC-SIGN, but also of heparan sulfate on the surface of MDCC (Fig. S3). Various viruses for which specific cellular receptors have been identified are known to use HSPGs as primary attachment receptors [64]. In particular, both HSPGs and DC-SIGN were shown to be required for attachment of HIV-1 and HSV-1 to dendritic cells [65], [66]. As such, HSPGs may serve as initial attachment factors to concentrate DENV on the cell surface and sulfated K5 derivatives may impair this initial cell attachment step. Alternatively (or additionally), the antiviral activity in dendritic cells may be caused by the capacity of these rather bulky compounds to sterically impair the interaction of DENV with DC-SIGN.

In either case, one would expect a comparable antiviral activity of both highly sulfated compounds in dendritic cells, as demonstrated in endothelial cells. However, whereas we found K5-OS(H) to be almost 10-fold more active than K5-N,OS(H) against DENV infection in dendritic cells, K5-N,OS(H) was shown to be ∼20- and 80-fold more efficient than K5-OS(H) against HIV-1 and HSV-1, respectively [45]. Thus, although K5-OS(H) and K5-N,OS(H) are structurally very similar and contain a comparable degree of sulfation, the specific arrangement of sulfate groups along the sugar chain is sufficient to elicit a different biological activity.

### K5-OS(H) and K5-N,OS(H) interfere with the early steps of DENV-2 infection

**Figure 7:**
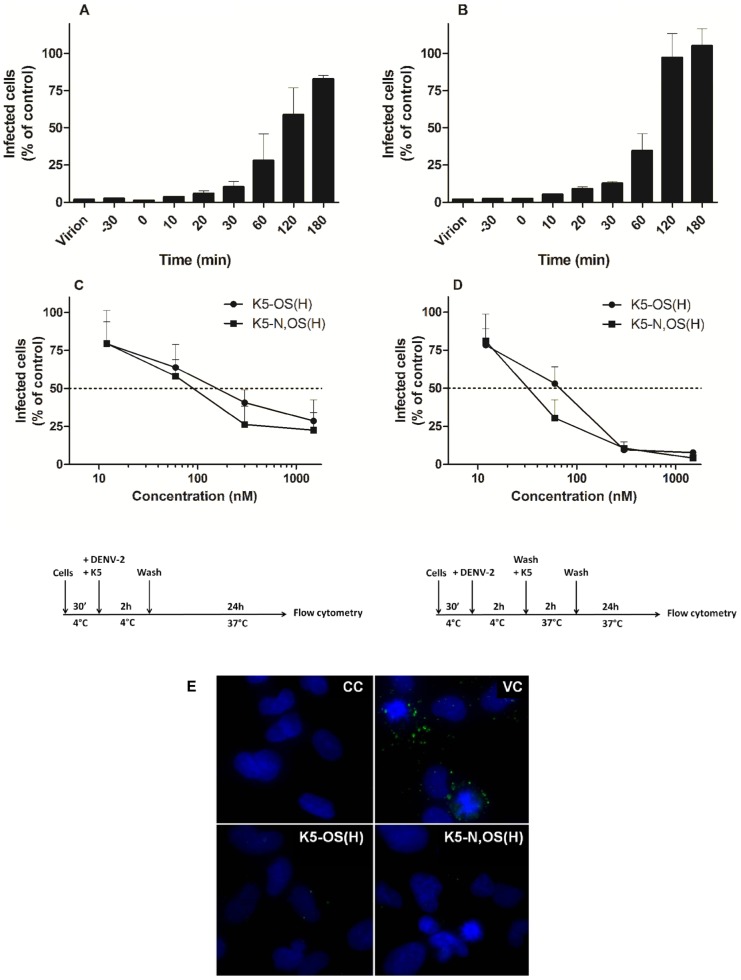
K5 derivatives inhibit the early steps of DENV infection in microvascular endothelial cells. K5-OS(H) (A) and K5-N,OS(H) (B) (500 nM) were added prior to (–30 min), together with (0 min), or at different times post-infection. The compounds were also incubated with virus inoculum for 30 min at 37°C before infection (virion). Viral infectivity was quantified after 24 h by flow cytometry using an anti-DENV-2 specific antibody. The percentage of infected cells relative to the virus control and standard deviation of 3 independent experiments are shown. Effect of K5-OS(H) and K5-N,OS(H) on DENV-2 attachment (C). HMEC-1 cells were incubated during 2 h at 4°C with DENV-2 in the presence or absence of various concentrations of compound. Next, cells were washed and after 24 h infection was analyzed by flow cytometry. Effect of K5-OS(H) and K5-N,OS(H) on DENV-2 entry (D). DENV-2 was allowed to attach to HMEC-1 cells for 2 h at 4°C. Unbound virus was washed away, various concentrations of compounds were added and temperature was raised to 37°C for 2 h to allow virus entry. Cells were washed and after 24 h infection was analyzed by flow cytometry. The percentage of infected cells relative to the virus control and standard deviation of 3 independent experiments are shown. Immunofluorescent staining of DENV-2 attachment to HMEC-1 cells (E). HMEC-1 cells were incubated with DENV-2 in the presence or absence of K5 derivatives (1.5 µM) on ice. Next, the nuclei (blue) and DENV-2 particles (green) were stained. K5-OS(H) and K5-N,OS markedly reduced DENV-2 attachment to HMEC-1 cells. CC: cell control, VC: virus control.

In order to determine at which stage of infection the highly sulfated K5 derivatives exert their antiviral activity, time-of-drug-addition experiments were performed. As shown in Fig. 7, pre-incubation of the virus inoculum with 0.5 µM of K5-OS(H) or K5-N,OS(H) for 30 min before infection almost completely suppressed DENV-2 infection of HMEC-1 cells. Similarly, when the compounds were added 30 min prior to, or at the time of infection, complete protection was observed. The inhibitory activity of the compounds steadily diminished when added at later time points after infection, i.e. from >85% and 65% inhibition when added at 30 min or 1 h after infection, respectively, to complete loss of activity when added 2-3 h post infection. Both highly sulfated K5 derivatives failed to display any inhibitory activity when allowed to interact with the HMEC-1 cell surface for 2 h, and subsequently washed away before infection of the cells (data not shown). These data indicate that K5-OS(H) and K5-N,OS(H) do not bind to cellular surface molecules but rather interact with the DENV envelope protein by which the early steps of virus infection (attachment and/or entry) are inhibited.

Therefore, we investigated in more detail the effects of K5 derivatives on DENV attachment and entry. In a first set of experiments, HMEC-1 cells were incubated with DENV-2 and various concentrations of K5-OS(H) and K5-N,OS(H) for 2 h at 4°C, a condition that allows only virus attachment. Next, unbound virus and compound were washed away and cells were incubated for 24 h at 37°C. Both K5-OS(H) and K5-N,OS(H) prevented DENV-2 attachment to HMEC-1 cells (EC_50_ value of 163±101 nM and 129±77 nM for K5-OS(H) and K5-N,OS(H) respectively, Fig. 7C), while K5 proved to be inactive (data not shown). Consistent with these results, immunofluorescence staining of HMEC-1 cells, incubated with DENV-2 in the presence or absence of the compounds for 1 h at 4°C showed that K5-OS(H) and K5-N,OS(H) markedly reduce DENV-2 attachment to HMEC-1 cells (Fig. 7E).

To determine the effect of K5 derivatives on viral entry, DENV-2 was allowed to attach to HMEC-1 cells at 4°C for 2 h. Next, unbound virus was washed away, different concentrations of compounds were added and the temperature was shifted to 37°C for 2 h to allow viral entry. Under these conditions, K5-OS(H) and K5-N,OS(H) retained their antiviral activity and even displayed a slightly higher activity than in the standard antiviral assay (EC_50_ values of 84±40 nM and 40±14 nM against virus entry versus 113±80 and 111±50 nM in the standard assay for K5-OS(H) and K5-N,OS(H), respectively, Fig. 7D), whereas K5 remained inactive (data not shown).

These data indicate that highly sulfated K5 derivatives inhibit DENV-2 infection of HMEC-1 cells by interfering with both viral attachment and entry. Similarly, K5-OS(H) and K5-N,OS(H) were shown to exert a post-attachment inhibitory activity against HIV, HSV-1 and HPV [40]–[42], whereas 100-fold higher drug concentrations were required to inhibit HCMV entry as compared to virus adsorption [43].

### *E. coli* K5 derivatives inhibit DENV infection by interacting with the viral envelope (E) protein

**Figure 8:**
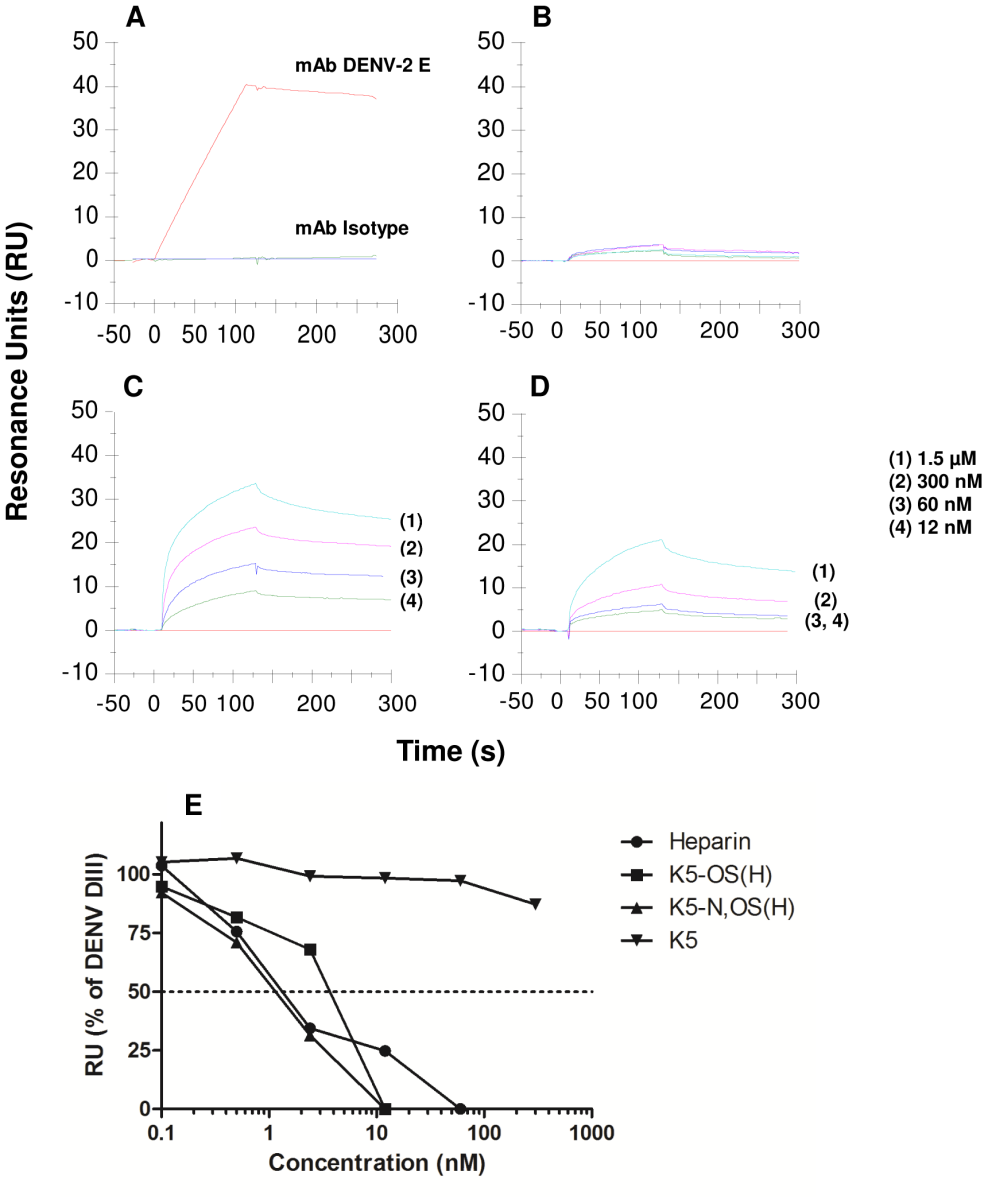
Surface plasmon resonance (SPR) analysis of the interaction between K5 derivatives and the DENV envelope protein. Sensorgrams show the binding of anti-DENV E antibody (positive control) and isotype antibody (negative control) (A), K5 (B), K5-OS(H) (C) and K5-N,OS(H) (D) to immobilized DENV E domain III. The binding curves of 0 to 120 s show the association, whereas those of 120 to 300 s show the dissociation phase. The y axis indicates the resonance signal as shown in resonance units (RU). Binding of DENV E domain III to immobilized heparin in the presence of different concentrations of compound (E). Heparin, K5-OS(H) and K5-N,OS(H) dose-dependently inhibited the interaction of domain III with immobilized heparin, whereas K5 was ineffective. Shown are the RU (% of domain III binding to the heparin chip).

To evaluate whether the highly sulfated K5-OS(H) and K5-N,OS(H) prevent DENV attachment and entry by interacting with the viral envelope protein, surface plasmon resonance (SPR) was used. Domain III of the DENV E protein has been implicated in receptor binding and contains two putative GAG binding sites, which are assumed to be involved in cell surface GAG interaction [54], [67]. Therefore, in a first set of experiments, recombinant DENV-2 E protein (domain III) was covalently immobilized on a CM5 sensor chip. Five-fold serial dilutions of K5, K5-OS(H) and K5-N,OS(H) (range 12 to 1500 nM) were exposed to the immobilized protein. Figure 8 demonstrates that both K5-OS(H) and K5-N,OS(H) bind to E protein domain III in a concentration-dependent manner. K5 in contrast, which showed no antiviral activity, was unable to bind the E protein. For kinetic analysis two-fold serial dilutions of K5-OS(H) and K5-N,OS(H) were used. A 1∶1 Langmuir kinetic fit was applied to obtain the association rate constant k_a_, dissociation rate constant k_d_, and the dissociation constant K_D_. The interaction of K5-OS(H) and K5-N,OS(H) with DENV E protein was characterized by k_a_-values of 3.0×10^4^ and 2.8×10^4^ M^−1^s^−1^ and k_d_-values of 1.8×10^−3^ and 1.4×10^−3^ s^−1^ which result in comparable high-affinity binding (K_D_ of 59 nM and 50 nM for K5-OS(H) and K5-N,OS(H), respectively).

Next, to mimic the interaction of DENV with cell-associated HSPGs, biotinylated heparin was captured on a streptavidin sensor chip and the binding of DENV E domain III to heparin was evaluated in the presence or absence of K5 derivatives. As shown in Fig. 8E, the binding of DENV E domain III to captured heparin was dose-dependently inhibited when domain III was injected together with heparin, K5-OS(H) or K5-N,OS(H). A complete inhibition of domain III binding was obtained in the presence of 60 nM of heparin or 12 nM of K5-OS(H) or K5-N,OS(H). In contrast, K5 was unable to abrogate the interaction of domain III with the heparin chip when administered at concentrations up to 300 nM (Fig. 8). Thus, both assays indicate that, by occupying the GAG binding sites on the viral E protein, K5-OS(H) and K5-N,OS(H) may prevent the initial attachment step (and subsequent entry) of the virus to endothelial cells.

In conclusion, our results indicate that DENV-2 infection of microvascular endothelial cells critically depends upon the interaction of HSPGs with the viral envelope protein. This interaction can be inhibited by natural GAGs or by highly sulfated *Escherichia coli* K5 derivatives. These compounds are devoid of anticoagulant activity, not cytotoxic and able to inhibit DENV attachment and entry. Thus, K5 derivatives represent a new class of anti-DENV antivirals that, *via* their inhibitory activity in endothelial and dendritic cells, may inhibit the cytokine storm that is thought to induce vascular leakage in DHF/DSS. Pharmacokinetic and bioavailability studies should be carried out to determine the potential of these compounds as anti-DENV agents.

## Supporting Information

Figure S1 **Infection of endothelial cells by DENV.** HMEC-1 and HMVEC-d cells were treated with medium alone (A, E) or infected with DENV-2 at a MOI of 1, 2 or 4 (C, G). Viral infectivity was quantified 24 h after infection by flow cytometry using an anti-DENV specific antibody (full histogram). Alternatively, HMEC-1 (D) and HMVEC-d (H) cells were infected with DENV-2 at a MOI of 1 and viral infectivity was quantified by flow cytometry after 24, 48 or 72 h post-infection. Background signal was determined by staining the cells with secondary PE-conjugated antibody only (B, F).
(TIF)

Figure S2 **The expression of β3 integrin on HMEC-1 cells was measured by flow cytometry (A).** Shown is the surface expression of the specific marker (full histogram) and background in the absence of primary antibody (dashed line). HMEC-1 cells were pre-treated with a functional blocking antibody against β3 integrin (B) or Cilengitide (C) and infected with DENV-2 at a MOI 1. Viral infectivity was quantified 24 h after infection by flow cytometry using an anti-DENV-2 specific antibody.
(TIF)

Figure S3 **Expression of heparan sulfate (A) and DC-SIGN (B) on MDDC.** Shown is the surface expression of the specific marker (full histogram) and background in the absence of primary antibody (dashed line).
(TIF)
