# Supplementary figures and images for: Sulfated Escherichia coli K5 Polysaccharide Derivatives Inhibit Dengue Virus Infection of Human Microvascular Endothelial Cells by Interacting with the Viral Envelope Protein E Domain III
Source: PLoS One. 2013 Aug 28;8(8):e74035. doi: 10.1371/journal.pone.0074035 (PMC3755990; doi:10.1371/journal.pone.0074035)

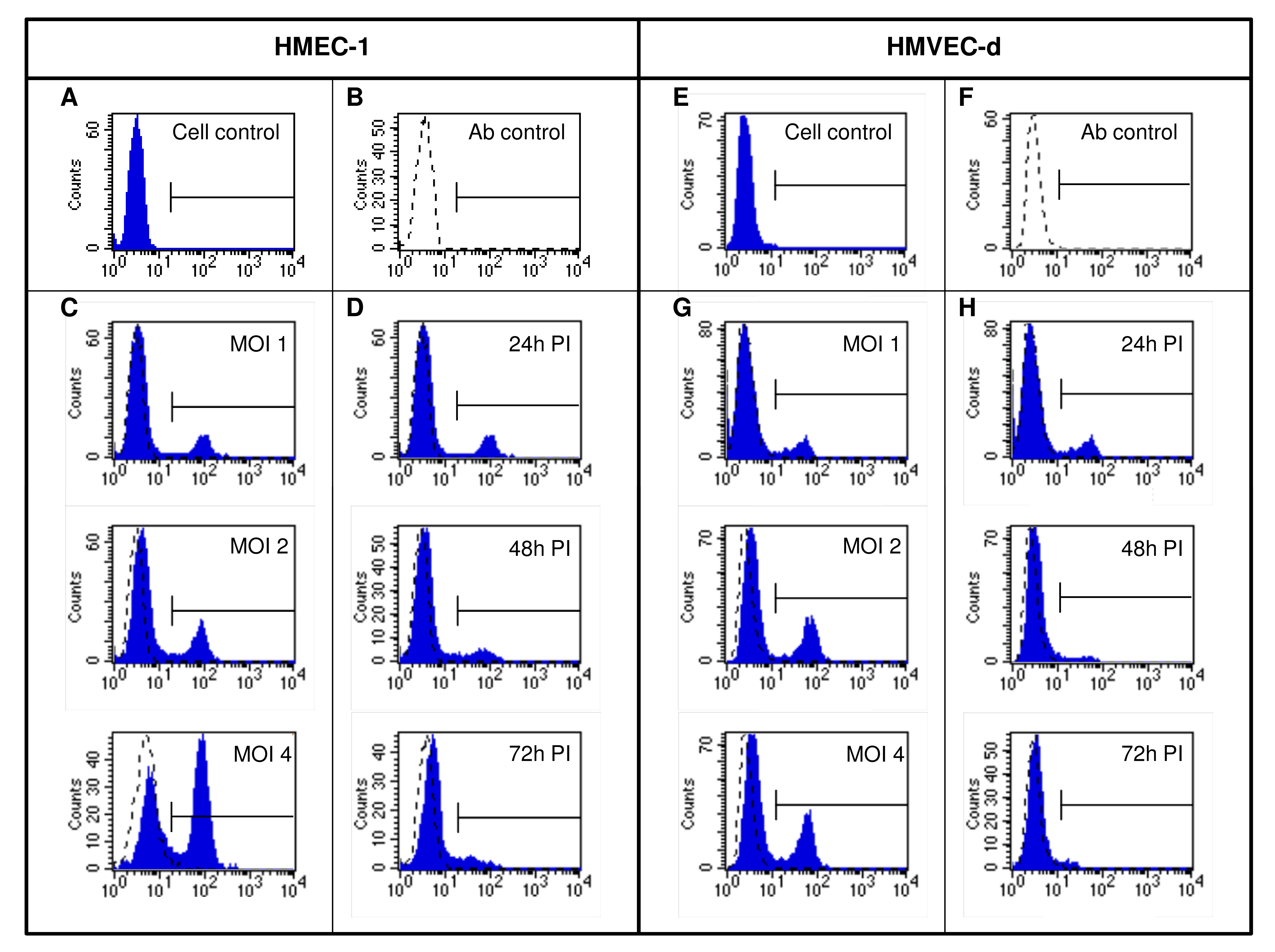

Supplement: Figure S1 — Infection of endothelial cells by DENV. HMEC-1 and HMVEC-d cells were treated with medium alone (A, E) or infected with DENV-2 at a MOI of 1, 2 or 4 (C, G). Viral infectivity was quantified 24 h after infection by flow cytometry using an anti-DENV specific antibody (full histogram). Alternatively, HMEC-1 (D) and HMVEC-d (H) cells were infected with DENV-2 at a MOI of 1 and viral infectivity was quantified by flow cytometry after 24, 48 or 72 h post-infection. Background signal was determined by staining the cells with secondary PE-conjugated antibody only (B, F). (TIF) [file pone.0074035.s001.tif]

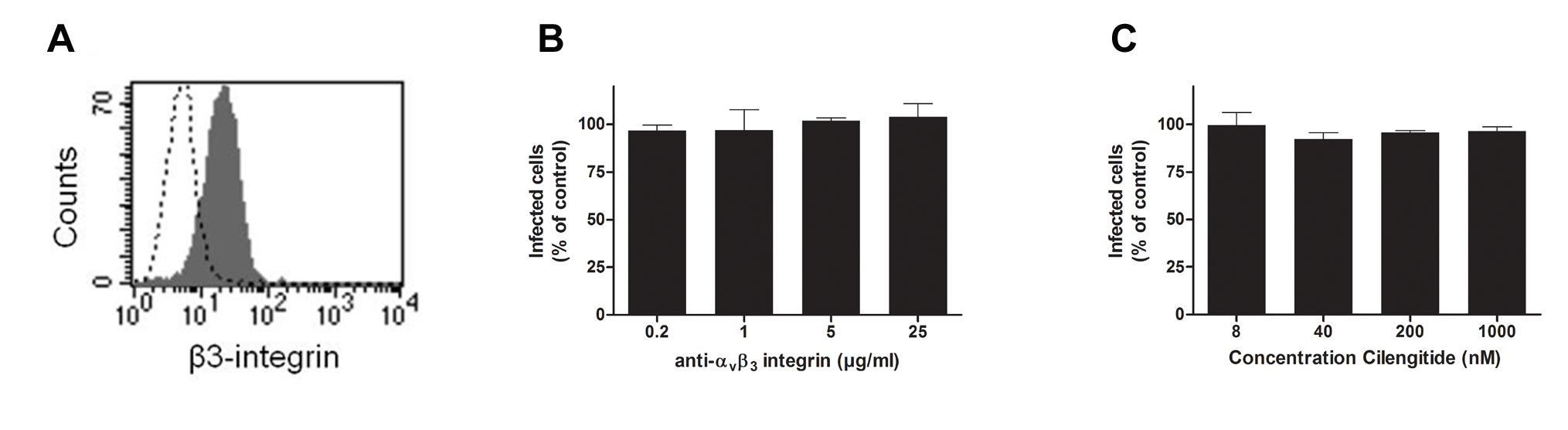

Supplement: Figure S2 — The expression of β3 integrin on HMEC-1 cells was measured by flow cytometry (A). Shown is the surface expression of the specific marker (full histogram) and background in the absence of primary antibody (dashed line). HMEC-1 cells were pre-treated with a functional blocking antibody against β3 integrin (B) or Cilengitide (C) and infected with DENV-2 at a MOI 1. Viral infectivity was quantified 24 h after infection by flow cytometry using an anti-DENV-2 specific antibody. (TIF) [file pone.0074035.s002.tif]

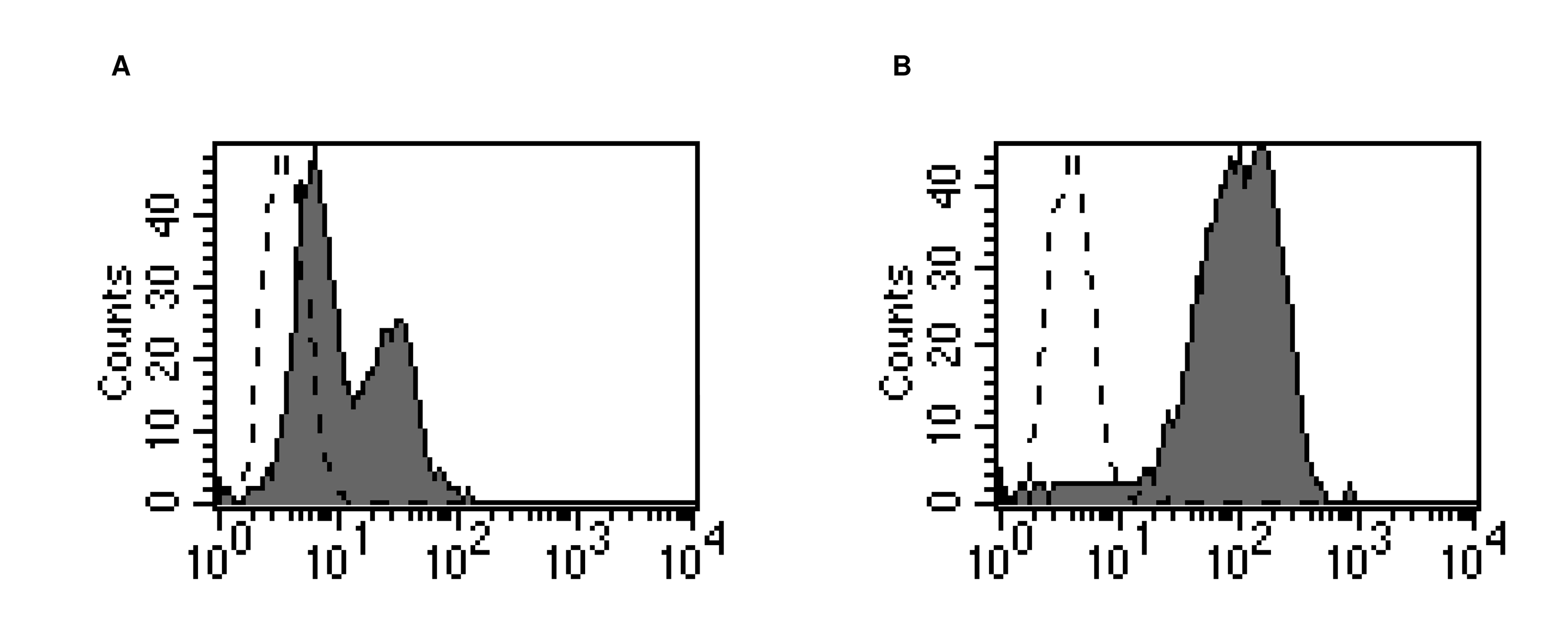

Supplement: Figure S3 — Expression of heparan sulfate (A) and DC-SIGN (B) on MDDC. Shown is the surface expression of the specific marker (full histogram) and background in the absence of primary antibody (dashed line). (TIF) [file pone.0074035.s003.tif]
